# Comparison of Respiratory Pathogen Detection in Upper versus Lower Respiratory Tract Samples Using the BioFire FilmArray Respiratory Panel in the Immunocompromised Host

**DOI:** 10.1155/2018/2685723

**Published:** 2018-04-05

**Authors:** Natalya Azadeh, Kenneth K. Sakata, Ali Saeed, John J. Mullon, Thomas E. Grys, Andrew H. Limper, Matthew J. Binnicker

**Affiliations:** ^1^Department of Pulmonary and Critical Care Medicine, Mayo Clinic, Rochester, MN, USA; ^2^Department of Laboratory Medicine and Pathology, Mayo Clinic, Rochester, MN, USA; ^3^Division of Clinical Microbiology, Mayo Clinic, Scottsdale, AZ, USA

## Abstract

**Background:**

The FilmArray Respiratory Panel (FARP) (BioFire Diagnostics, Inc.) is a multiplex, polymerase chain reaction (PCR) technique that can detect 17 respiratory viruses and 3 bacterial targets in a single reaction. Immunocompromised hosts (ICH) with respiratory illnesses often undergo bronchoscopy with bronchoalveolar lavage (BAL). This prospective study aimed to evaluate the yield and concordance of NP and BAL FARP testing when performed on the same patient concurrently.

**Methods:**

From February to December 2016, 125 patients (100 ICH and 25 non-ICH) were enrolled. NP swabs and BAL samples were sent for FARP testing.

**Results:**

The yield of the BAL FARP among ICH and non-ICH was 24% (24/100) and 8% (2/25), respectively. The yield of positive NP swabs in ICH was 27% (27/100) versus 4% (1/25) in non-ICH. The majority of patients (89%; 111/125) had concordant results between NP and BAL specimens. Of the 24 ICH patients who had a positive BAL FARP, the majority (79%) had the same pathogen detected from the NP swab.

**Conclusion:**

The FARP may be useful in the ICH. Given the high concordance, in patients whom a pathogen is identified on the NP FARP, a FARP performed on BAL will likely yield the same result. However, if the NP FARP is negative, performing the test on a BAL sample may have an incremental yield.

## 1. Introduction

The FilmArray® Respiratory Panel (FARP) (BioFire Diagnostics, Salt Lake City, UT) is a multiplex PCR assay that can rapidly (∼60 min) detect 17 common respiratory viruses and 3 bacterial targets in a single reaction [1]. Published studies have shown that multiplex PCR panels are more rapid and sensitive compared to routine culture and antigen detection methods [1–8]. Although multiplex panels are rapid and accurate, the high cost to the laboratory and patient may limit their use in resource-limited settings.

For the majority of immunocompetent patients with respiratory symptoms, diagnostic testing may not be required. If testing is completed, it is usually done on an easily accessible specimen type (e.g., throat swab or nasopharyngeal [9] (NP) swab) and is generally limited to targeted pathogens (e.g., influenza virus). However, in patients who are immunocompromised or critically ill, a rapid, syndromic panel may be of benefit. Furthermore, patients with persistent or progressive symptoms may require a more extensive diagnostic evaluation, including bronchoscopy with collection of bronchoalveolar lavage (BAL) fluid. The utility of FARP testing using NP swabs and BAL specimens collected from immunocompromised hosts (ICH) has been investigated [2, 7, 10]. Our group completed a previous, retrospective study analyzing the FARP in patients who underwent bronchoscopy with BAL for evaluation of a respiratory illness. The results of the multiplex panel on BAL were compared to those of testing on a NP swab that had been collected within 7 days of the bronchoscopy. This study demonstrated high concordance (77%) between the FARP completed on NP swabs and BAL specimens [10]. However, the prior study was limited by the fact that the NP swab and BAL fluid were collected at different time points, and the data were analyzed mainly to compare overall concordance of the FARP between the two specimen types. In order to extend on these prior observations, we sought to prospectively evaluate the yield and concordance of the FARP using NP swabs and BAL fluid collected concurrently from ICH and non-ICH comparison group. This study was approved by the Mayo Clinic Institutional Review Board.

## 2. Methods

### 2.1. Patient Population

Between February and December 2016, 125 patients (100 ICH and 25 non-ICH) who underwent a bronchoscopy with BAL as part of their routine clinical care were enrolled in this study. Ages ranged between 26 and 85 years (mean 70 years), and 58% were male. An immunocompromised status was defined as patients who were (1) recipients of systemic steroids (for ≥21 days), disease-modifying agents (e.g., inhibitors of tumor necrosis factor), chemotherapy, or calcineurin inhibitors, (2) HIV positive, (3) solid organ or stem cell transplant recipients, and/or (4) positive for a lymphoproliferative or myelodysplastic syndrome (Table 1). ICH patients (e.g., lung transplant recipients) who were undergoing routine graft rejection surveillance transbronchial biopsies and BAL were also included in our analysis. They would also serve as an ICH control group to determine whether the FARP tested positive in asymptomatic ICH patients. Twenty-five patients who did not meet the criteria listed above were also included as part of a non-ICH comparison group. These were patients undergoing bronchoscopy with BAL to rule out infection but were not considered immunocompromised. Once enrolled, the patients' electronic medical records were reviewed and the following information was obtained: demographics (age and sex), presence and reason of immunosuppression, microbiology testing data from the BAL specimen (e.g., cultures and PCR), specifics of the respiratory illness (e.g., cough and wheezing), and other clinical or radiographic features. When the results of other microbiology studies (e.g., routine culture) were reviewed, only those results that led to an impact on patient management and were considered to be true pathogens were analyzed.

### 2.2. Specimen Collection

The protocol for collection and testing of specimens was approved by the institutional review board at our center. Patients provided informed consent and subsequently were sedated prior to bronchoscopy. Following sedation, a wire-shafted, BBL CultureSwab (Becton Dickinson, Franklin Lakes, NJ) was used to sample the posterior nasopharynx per routine protocol and subsequently placed in 3 mL of M5 viral transport media (Remel, Lenexa, KS). Next, a bronchoscopy with BAL was performed, in which 20 mL aliquots of normal saline were instilled into a subsegmental bronchus corresponding to an area of radiographic abnormality on CT. Forty mL of BAL fluid was recovered by immediate return suction. A 1-2 mL aliquot of the BAL specimen and the NP swab were transported to the laboratory, stored at 4°C, and tested within 24 hours.

### 2.3. Routine Microbiology Testing

In addition to FARP testing, which was performed solely for study purposes, the following microbiology studies were completed on the BAL sample as part of each patient's routine clinical care: Gram stain, fungal smear, acid-fast smear, routine cultures (bacterial, fungal, viral, and mycobacterial), real-time PCR for influenza, respiratory syncytial virus (RSV), *Pneumocystis jirovecii* (PJP), adenovirus, and *Legionella* spp. The NP swab was collected solely for study purposes, and no other microbiology studies were conducted on this source.

### 2.4. FilmArray Respiratory Panel Testing

NP swabs in viral transport media were tested by the FilmArray Respiratory Panel according to the manufacturer's instructions. In addition, 0.3 mL of raw BAL fluid was loaded into a FilmArray v1.6 pouch and tested on the FilmArray 2.0 instrument (BioFire) following the same protocol used for NP swabs.

### 2.5. Statistical Analysis

All statistical analysis was done using comparison of proportions online chi-squared calculator (https://www.medcalc.org/calc/comparison_of_proportions.php).

## 3. Results

Among 125 patients enrolled in this study, the multiplex panel was positive for at least one target in 28 (22%) NP specimens and 26 (21%) BAL samples. The majority (89%; 111/125) of paired NP and BAL samples had concordant results. Of these, 73% (91/125) were negative for all targets in both NP and BAL specimens. Sixteen percent (20/125) of paired NP and BAL samples had the same pathogen(s) detected by the FARP, while the remaining 11% (14/125) had discordant results in NP and BAL specimens by the FARP (Table 2). Rhinovirus/enterovirus was the most frequently detected pathogen, being identified in 15.2% (19/125) of NP swabs and 11.2% (14/125) of BAL specimens. In ICH patients, the overall yield of the FARP was higher in both NP and BAL samples compared with non-ICH patients (*p*=0.02) (Tables 3 and 4). Twenty-seven (27%) of 100 ICH patients had at least one pathogen detected in the NP swab. Of these, 19/27 (70%) had the same pathogen(s) detected from the paired BAL specimen. The remaining 8 (30%) patients had a negative result in the paired BAL sample. The FARP was positive for at least one pathogen in 24 (24%) BAL specimens collected from ICH patients, and of these, 19/24 (79%) had the same pathogen detected in the paired NP swab sample. The remaining 5 (21%) patients had a negative result in the NP specimen (Table 3).

In comparison with the FARP, the overall yield of other microbiology studies (e.g., routine bacterial, fungal and viral culture, and individual real-time PCR studies) on the BAL specimens was 29% (36/125) (Table 5). In the ICH group, at least one microbiology study (not including FARP) was positive in 25% (25/100) of the BAL samples, compared to 24% (24/100) of BAL specimens being positive by the FARP. Among 32 ICH patients who had either a positive NP or BAL FARP, 10 (31%) had additional yield from routine (non FARP) microbiology studies. Among 68 ICH patients who had negative FARP results from both the NP swab and BAL, 16 (24%) had an organism identified by at least one of the routine microbiology tests (Table 5). In non-ICH patients, other microbiology studies were positive for at least one pathogen in 11/25 (44%) BAL specimens. The majority (92%; 23/25) of these patients had negative FilmArray results in both NP and BAL samples (Table 5).

In the ICH group, the most common indication for bronchoscopy with BAL in our study was a radiographic infiltrate(s) on chest imaging (*n*=83), followed by a diagnosis of pneumonia (*n*=24), cough (*n*=22), sepsis (*n*=21), hypoxia (*n*=14), dyspnea (*n*=10), upper respiratory tract infection (*n*=6), or FEV1 (forced expiratory volume in 1 second) decline (*n*=2). The majority of patients had a clinical and/or radiographic indication for bronchoscopy. Seventy-two (72%) ICH patients and 21 (84%) non-ICH patients had both radiographic and clinical indications for BAL. Ten patients underwent bronchoscopy/BAL for routine surveillance after lung transplant. In one of these patients, human rhinovirus/enterovirus was detected from both the NP and BAL samples. When these surveillance patients (without a clinical or radiographic indication for BAL) were excluded from analysis, the yield of the FARP increased from 27% (27/100) to 28.8% (26/90) in NP swabs and from 24% (24/100) to 25.5% (23/90) in BAL specimens. Out of the 100 ICH patients, 78 had a clinical indication for bronchoscopy/BAL (72 of these also had a radiographic indication) and 22 patients had no clinical indication for bronchoscopy (12/22 patients had only radiographic indications and 10/22 patients underwent bronchoscopy for routine surveillance after lung transplant). Those patients with a clinical indication for bronchoscopy/BAL were more likely to have a positive FARP result from either the NP or BAL sample compared with those that had no clinical indication for BAL (36% versus 18%, *p*=0.018). Among six patients that had clinical symptoms but no radiographic features of respiratory disease, FilmArray was positive in 3/6 (50%) patients in either the NP swab or BAL samples. Furthermore, the yield of the FARP was 34.7% (25/72) in patients with both clinical and radiographic indications for bronchoscopy/BAL versus 25% (3/12) in patients with a radiographic indication alone.

## 4. Discussion

The results of this study suggest that the FARP may be useful in the evaluation of ICH with suspicion of lower respiratory tract infection, as the yield of this test was higher in ICH (32%) compared to non-ICH patients (8%) (*p*=0.02) (Tables 2 and 3). Twenty-seven ICH patients (27%) had a positive NP FARP, with 19/27 (70%) having the same pathogen(s) detected from the paired BAL sample. The remaining 8 (30%) patients had a negative FARP result in BAL fluid (Table 2). This suggests that if a pathogen is detected from the NP swab by the FARP, performing the multiplex assay on BAL fluid is unlikely to provide additional yield for pathogens included on the FARP. Among the ICH group, 24/100 (24%) had a pathogen detected from BAL fluid by the FARP. The majority (79%; 19/24) of these patients had the same pathogen(s) identified from the NP specimen, while 5 (21%) had a negative FARP result on the NP swab (Table 2). We conclude that if the FARP is negative on a NP swab, there may be additional value of testing BAL fluid by the multiplex assay. It should be noted that regardless of the FARP results on an NP swab, proceeding with bronchoscopy/BAL may still be indicated in ICH patients with suspected infection since the FARP does not test for certain pathogens (e.g., *Aspergillus* spp., *Pneumocystis*, *Nocardia* spp., and *Mycobacteria* spp.) that can be important causes of disease in the immunosuppressed population. In support of this, in our cohort, those with a positive FARP on either NP or BAL samples had an additional pathogen(s) detected by routine microbiology studies in 31% of cases. Furthermore, among those patients with negative FARP results on both NP and BAL specimens, there was an additional yield from routine microbiology studies on the BAL fluid in 23.5% of cases (Table 5).

The yield of routine microbiology testing (e.g., cultures and individual real-time PCR assays) in the ICH cohort was 25%, compared to the FARP being positive in 27% of NP swabs and 24% of BAL specimens. These results suggest that the FARP assay may serve as a rapid alternative to routine testing for those targets that are included on the multiplex panel. Furthermore, FARP testing may play an important role in patient management decisions, including infection control and antimicrobial stewardship [11]. If a bacterial pathogen is detected, broad-spectrum coverage could be modified to offer more specific antimicrobial therapy. In contrast, if a viral pathogen is detected and bacterial cultures remain negative, it may be prudent to discontinue antibiotics and initiate targeted antiviral therapy [11]. Bronchoscopy with BAL is routinely ordered in ICH patients at our institution. Our study showed that standard microbiology studies on BAL fluid had a yield of 25% in this cohort, which is comparable to the yield of the FARP (24%). We believe that the comparable yield of FARP in this population provides additional support for this noninvasive test as a reasonable adjunct to BAL testing in the ICH.

Many ICH patients with respiratory illness are placed on empiric, broad-spectrum antimicrobial therapy before the BAL is collected, which can confound the yield of BAL cultures. Multiplex molecular testing, however, may not be affected by the prior administration of antibiotics. We propose that when used in conjunction with routine microbiology studies, FARP testing on BAL fluid may have a significant impact on the diagnosis and management of ICH patients with respiratory illnesses.

Conversely, FARP appears to be less useful in non-ICH patients. In our study, the yield of routine microbiology studies in non-ICH patients was higher than that in ICH patients (44% versus 25%, resp.). However, the FARP showed a lower yield when compared to routine cultures in the non-ICH cohort (8% versus 32%, resp.).

The performance of the FARP observed in our study is comparable with other studies in the literature. Our group previously performed a retrospective review of FARP testing on NP and BAL samples collected within one week of each other. This study reported the FARP to be positive in 20.9% (18/86) of NP swabs versus 37.2% (32/86) of BAL specimens [10]. The higher yield from BAL is likely due to the fact that only patients with a radiographic and/or clinical indication for BAL were included.

Hammond et al. [2] also assessed the performance of the FARP using NP swabs (*n*=34) and BAL specimens (*n*=56) collected from ICH patients (*n*=87). Importantly, this study did not compare NP swabs and BAL fluid that were collected concurrently. The results suggested that the FARP had an overall yield of 29% and identified more respiratory pathogens compared to conventional methods (direct fluorescent antibody (DFA), enzyme immunoassays, and viral culture) [2].

Soccal et al. [7] assessed the detection of respiratory viruses by individual real-time PCR assays using NP and BAL samples collected concurrently from lung transplant patients. They observed an overall viral positivity rate of 29.3% in NP samples and 17.2% in BAL samples. They also demonstrated that lung transplant patients had a much slower lung function recovery if they were found to have evidence of rejection in addition to viral pathogen detection, providing important prognostic information [7]. The lower yield of PCR in BAL specimens could be explained by the inclusion of any lung transplant patient that underwent bronchoscopy, without necessarily having respiratory symptoms, pulmonary infiltrates, or decline in airflow. Our data supports the notion that those without clinical or radiographic indications for BAL have a lower rate of pathogen detection.

This study has several limitations that should be discussed. First, the number of NP (*n*=28) and BAL (*n*=26) samples testing positive by the FARP was relatively low, so this limits the conclusions that can be made regarding specific pathogens tested. Second, due to the heterogeneous radiographic and clinical findings in these patients, it was difficult to identify specific clinical or radiologic features that would prompt the performance of FARP testing of BAL fluid versus a NP swab alone. Despite these limitations, this is the first study to compare the performance of the FARP using BAL fluid and NP swabs collected concurrently from ICH patients and a non-ICH comparison group.

When the FARP is performed in conjunction with routine microbiology tests in the ICH, it can provide important diagnostic information, especially in patients with clinical manifestations of respiratory illness. Our data suggest that, in this cohort, FARP testing should be initially performed on a NP swab. If positive, there is limited additional yield of testing the FARP on BAL fluid. However, if the results of the multiplex assay are negative on the NP swab, testing a BAL specimen may provide additional information, especially in patients with lower respiratory tract disease. It is necessary to highlight that the FARP does not cover all causes of respiratory infection, and therefore, routine microbiology testing (e.g., bacterial, mycobacterial, and fungal cultures) will continue to be essential.

In conclusion, respiratory infections that are generally well tolerated in the immunocompetent host can be serious and life-threatening in the ICH. The early detection, diagnosis, and appropriate management of these infections can have a substantial impact on clinical care and outcomes. Therefore, multiplex testing may play an important role in the management of ICH presenting with respiratory symptoms.

## Figures and Tables

**Table 1:** Reason for immunocompromised status.

Condition leading to immunosuppression	Number (%) of patients
HSCT	49 (49.0)
SOT	27 (27.0)
Connective tissue disease on immunosuppression	10 (10.0)
Chronic prednisone therapy	8 (8.0)
Cytotoxic chemotherapy	3 (3.0)
HIV/AIDS	2 (2.0)
CVID	1 (1.0)
Total	100 (100.0)

HSCT, hematopoietic stem cell transplant recipient; SOT, solid organ transplant recipient; CVID, common variable immune deficiency.

**Table 2:** Concordance of the FilmArray Respiratory Panel when performed on paired NP swabs and BAL fluid specimens (*n*=125).

Number (%) of specimens yielding concordant FilmArray results	
NP and BAL both positive	20 (16.0%)
NP and BAL both negative	91 (72.8%)
Total	111 (88.8%)
*Number (%) of specimens yielding discordant FilmArray results*	
NP positive, BAL negative	8 (6.4%)
NP negative, BAL positive	6 (4.8%)
Total	14 (11.2%)

BAL, bronchoalveolar lavage; NP, nasopharyngeal.

**Table 3:** Comparison of FilmArray Respiratory Panel using paired nasopharyngeal swab and BAL samples collected from immunocompromised hosts (*n*=100).

Number (%) of NP swab samples with a FilmArray result	Number (%) of BAL specimens with a FilmArray result	
	Positive	Negative
Positive	19 (19.0)^a^	8 (8.0)^b^
Negative	5 (5.0)^c^	68 (68.0)

BAL, bronchoalveolar lavage; NP, nasopharyngeal; ^a^rhinovirus/enterovirus (*n*=9), parainfluenza type 3 (*n*=2), coronavirus HKU1 (*n*=1), coronavirus NL63 (*n*=1), parainfluenza type 1 (*n*=1), human metapneumovirus (*n*=1), *Bordetella pertussis* (*n*=1), influenza A H3 (*n*=1), coronavirus OC43 and rhinovirus/enterovirus (*n*=1), and adenovirus, coronavirus 229E, and rhinovirus/enterovirus (*n*=1); ^b^rhinovirus/enterovirus (*n*=6) and coronavirus HKU1 (*n*=2); ^c^rhinovirus/enterovirus (*n*=2), coronavirus HKU1 (*n*=1), parainfluenza type 2 (*n*=1), and respiratory syncytial virus (*n*=1).

**Table 4:** Comparison of FilmArray respiratory panel using paired nasopharyngeal swab and BAL samples collected from immunocompetent hosts (*n*=25).

Number (%) of NP swab samples with a FilmArray result	Number (%) of BAL specimens with a FilmArray result	
	Positive	Negative
Positive	1 (4.0)^a^	0 (0.0)
Negative	1 (4.0)^b^	23 (92.0)

BAL, bronchoalveolar lavage; NP, nasopharyngeal; ^a^rhinovirus/enterovirus (*n*=1); ^b^codetection of coronavirus OC43, parainfluenza type 1, and respiratory syncytial virus (*n*=1).

**Table 5:** Yield of routine microbiology studies in bronchoalveolar lavage fluid collected from immunocompromised (*n*=100) and immunocompetent (*n*=25) patients.

Patient group and FilmArray result	Total number	Number (%) of BAL fluid samples that were positive by a routine microbiology test^a^
ICH	100	25^b^ (25.0%)
Positive FARP	32	10 (31.3%)
Negative FARP	68	15 (23.5%)
Non-ICH	25	11^c^ (44.0%)
Positive FARP	2	1 (50.0%)
Negative FARP	23	10 (43.5%)

No., number; BAL, bronchoalveolar lavage; ICH, immunocompromised host; FARP, FilmArray Respiratory Panel; ^a^routine microbiology testing included Gram staining, fungal smear, acid-fast smear, routine cultures (bacterial, fungal, viral, and mycobacterial), real-time PCR for influenza A/B, respiratory syncytial virus (RSV), *Pneumocystis jirovecii*, adenovirus, and *Legionella* spp.; ^b^organisms recovered/detected: *Aspergillus* spp. (*n*=7), cytomegalovirus (*n*=4), *Pseudomonas* spp. (*n*=3), *Klebsiella* spp. (*n*=2), *S. aureus* (*n*=2), methicillin-resistant *Staphylococcus aureus* (*n*=1), *Serratia marcescens* (*n*=1), *Pneumocystis jirovecii* (*n*=1), *Scopulariopsis* (*n*=1), *Bordetella bronchiseptica* (*n*=1), *Haemophilus influenzae* (*n*=1), *Stenotrophomonas maltophilia* (*n*=1), *Proteus mirabilis* (*n*=1), *Mycobacterium avium* complex (*n*=1), and *Histoplasma capsulatum* (*n*=1) (three patients had more than one pathogen detected); ^c^organisms recovered/detected: *Mycobacterium avium* complex (*n*=4), *Aspergillus* spp. (*n*=2), *Staphylococcus aureus* (*n*=2), *Stenotrophomonas maltophilia* (*n*=1), *Mycobacterium abscessus* group (*n*=1), *Achromobacter* spp. (*n*=1), *Haemophilus influenzae* (*n*=1), *Citrobacter* spp. (*n*=1), and *Klebsiella* spp. (*n*=1) (four patients had more than one pathogen detected).
